# Facultative and Obligate Trees in a Mesic Savanna: Fire Effects on Savanna Structure Imply Contrasting Strategies of Eco-Taxonomic Groups

**DOI:** 10.3389/fpls.2018.00644

**Published:** 2018-05-18

**Authors:** Michelle E. Freeman, Brett P. Murphy, Anna E. Richards, Peter A. Vesk, Garry D. Cook

**Affiliations:** ^1^School of BioSciences, The University of Melbourne, Parkville, VIC, Australia; ^2^CSIRO Land and Water, Tropical Ecosystems Research Centre, Winnellie, NT, Australia; ^3^Research Institute for the Environment and Livelihoods, Charles Darwin University, Darwin, NT, Australia

**Keywords:** savanna, fire frequency, fire trap, tree, size class distribution, woody plant strategies, escape

## Abstract

Fire is a major determinant of savanna tree communities and, as such, manipulation of fire frequency is an important management tool. Resolving the effects of fire management on tree size class distributions can help managers predict and plan for short-term ecological and economic outcomes, reveal different strategies by which woody plants cope with frequent fire, and help us predict vegetation changes under future fire scenarios. Savanna structure and size class distribution are strongly influenced by the ability of suppressed tree resprouts to escape stem death by frequent fire. A widespread assumption is that resprouts have an imperative to escape fire to reach sexual maturity in the canopy and thereby ensure long-term species viability. We use a census of Australian mesic savanna tree communities subjected to annual, triennial, and fire exclusion (unburnt) fire treatments to ask how fire frequency affects size class distributions within and between eco-taxonomic groups of species. Total tree densities did not significantly differ, but were highest in the triennial (7,610 ± se 1,162 trees ha^−1^) and unburnt fire treatments (7,051 ± se 578 trees ha^−1^) and lowest in the annual fire treatment (6,168 ± se 523 trees ha^−1^). This was caused by increased sapling densities in the triennial and unburnt fire treatments, predominantly of *Acacia* and pantropical genera. Eucalypts (*Eucalyptus* and *Corymbia* spp.) dominated the canopy across all fire treatments indicating relatively greater success in recruiting to larger sizes than other species groups. However, in the sub-canopy size classes eucalypts co-dominated with, and in some size classes were outnumbered by, pantropicals and *Acacia*, regardless of fire treatment. We hypothesize that such results are caused by fundamental differences in woody plant strategies, in particular sexual reproduction, that have not been widely recognized in Australian savannas.

## Introduction

There is little doubt that disturbance by fire is one of the most pervasive determinants of savanna tree communities (Bond and Van Wilgen, 1996; Scholes and Archer, 1997; Higgins et al., 2000; Baudena et al., 2010; Staver et al., 2011). Frequent fire regimes affect vegetation structure, mainly through suppression of saplings to the ground layer and death of susceptible canopy trees (Williams et al., 1999; Peterson and Reich, 2001; Higgins et al., 2007), and affect species composition, through preferencing functional types equipped to cope with frequent fire (Bond and Van Wilgen, 1996; Bond and Keeley, 2005; Silva and Batalha, 2010). Manipulation of fire regimes is therefore an important management tool for shaping savannas. For example, across northern Australia prescribed burning during the early dry season (April–July, inclusive), when fires tend to be of low intensity, is widely used to generate carbon credits (by reducing the amount of fuel burnt per unit area per year) and maximize biodiversity conservation (Douglass et al., 2011; Russell-Smith et al., 2013). An increasingly large area is being actively managed under this “savanna burning” paradigm for greenhouse gas emissions abatement (Russell-Smith et al., 2017), while recent studies have also aimed to quantify carbon sequestration potential of different fire management scenarios for incorporation into carbon accounting methodologies (Richards et al., 2012). In addition to maximizing carbon, fire management has a role to play in promoting shrub densities that may be critical to native fauna conservation (McGregor et al., 2015; Davies et al., 2017).

Within these contexts, it is highly relevant that we understand the impacts of fire regimes on tree community composition and structure. Understanding fire effects requires consideration of different tree growth strategies. According to (Gignoux et al., 2009, p. 494), “true savanna [tree] species” are those “that are able to both enter and exit the resprout stage and recruit as adult trees under the fierce disturbance regime of savannas.” This assertion assumes that escape from the fire trap of stem topkill followed by basal resprouting is necessary for trees to reach sexual maturity in the canopy and thereby maintain population viability (Bond and Van Wilgen, 1996; Murphy and Bowman, 2012). Quantifying fire frequency effects on savanna structure is therefore important not just from a carbon and conservation perspective, but because perpetual suppression of resprouts may prevent trees from reaching sexual maturity, affecting long-term population viability (Werner, 2012).

Tree resprouts are small (often ~1 m tall) multi-stemmed individuals that persist through frequent burning via basal resprouting (Bond and Midgley, 2001; Grady and Hoffmann, 2012). Fire frequency affects the rate at which resprouts can escape from the fire trap (in which stems are repeatedly topkilled by fire) to reach the canopy (e.g., see Fensham, 1990; Prior et al., 2010; Bond et al., 2012; Higgins et al., 2012). The fire trap causes a demographic bottleneck at the sapling stage because saplings topkilled by fire often cannot recover their pre-fire height, especially in short inter-fire periods, and thus regress to resprout size (Hoffmann and Solbrig, 2003; Prior et al., 2010; Freeman et al., 2017). With frequent fire, this demographic bottleneck results in multimodal tree size class distributions characterized by abundant resprouts, relatively few canopy trees, and a distinct lack of trees of sapling size (Hoffmann and Solbrig, 2003; Lehmann et al., 2009; Nguyen and Baker, 2016). With longer inter-fire periods we might expect saplings to recover their pre-fire height, resulting in reverse-J (exponential) size class distributions (Rubin et al., 2006) and if fire is excluded, large midstorey and canopy tree densities may eventually increase (Hoffmann, 1999; Woinarski et al., 2004). We therefore might expect tree resprout densities to decrease with decreasing fire frequency as transitions to larger size classes is facilitated. However, previous studies report that resprouts of different species vary in their response to fire frequency (Peterson and Reich, 2001; Russell-Smith et al., 2003; Higgins et al., 2007; Prior et al., 2010).

In Australian savannas, eucalypt vs. non-eucalypt eco-taxonomic groups are often used to differentiate species with different expected fire response (Werner, 2005; Prior et al., 2009; Bond et al., 2012). Eucalypts (*Eucalyptus* and *Corymbia* spp.), which tend to be tall-growing and thin-barked, appear better able to escape from the fire trap of both frequent and intense fire than species of pantropical affiliation. Although a diverse group of species, pantropicals are generally wider-growing, have thicker bark and are more likely to be suppressed by the fire trap than eucalypts (Lawes et al., 2011a,b; Bond et al., 2012). Regardless of fire regime, acacias and Proteaceae species have high individual turnover rates compared to other species (Prior et al., 2009). Lower fire frequencies have been shown to facilitate the transition of pantropical species into the midstorey, while eucalypt stand structure remains stable—calling into question the importance of fire to structuring eucalypt populations (Russell-Smith et al., 2003; Woinarski et al., 2004; Murphy et al., 2015).

In this study, we quantify the effect of different fire frequencies on the stand structure of an Australian mesic savanna tree community, classified by eco-taxonomic group. We use a census of tree populations subjected to three experimental fire treatments, implemented over 6 years, to ask two fundamental questions: (1) how does fire frequency affect tree densities within different size classes; and (2) how does fire frequency affect tree densities across size classes within and between eco-taxonomic groups of species? The fire treatments were implemented as part of the Tiwi Carbon Study (Richards et al., 2012) to evaluate the carbon sequestration potential of different management regimes as a basis for remote communities to participate in the carbon economy. Resolving fire management effects on tree size class distributions will help managers and modelers predict and plan for different ecological and economic outcomes.

## Methods

### Study area

Melville Island, the second largest island of Australia (5,790 km^2^, maximum elevation 102 m above sea level), is one of the Tiwi Islands, which lie 60 km north-east of Darwin in the Northern Territory of Australia. The climate is monsoonal with rainfall averaging 1,840 mm in the wet season (November–April) and 144 mm in the dry season (May–October; BOM, 2017). Approximately 77% of Melville Island is frequently burnt savanna co-dominated by *Eucalyptus tetrodonta, Eucalyptus miniata*, and *Corymbia nesophila* (Woinarski and Baker, 2002). The understorey is dominated by C4 grasses and multi-stemmed woody resprouts. The height (≤30 m) and canopy cover (~30–60% projected foliage cover) of this vegetation community reflects an open forest formation (Specht, 1970), but the prevalence of C4 grasses in the understorey, fire-tolerant tree species, and lack of midstorey driven by very frequent fire equates these open forests with savannas elsewhere (Ratnam et al., 2011).

**Figure 1:**
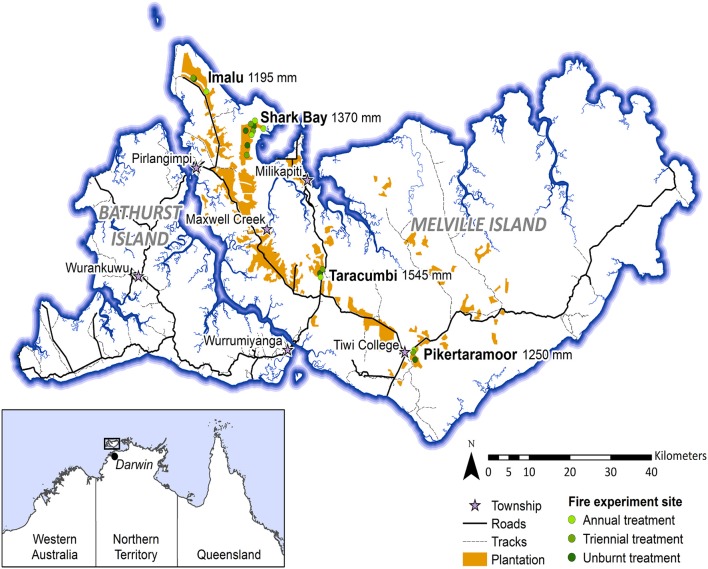
Map of the Tiwi Islands showing locations of the Tiwi Carbon Study fire treatment sites (Imalu, Shark Bay, Taracumbi, Pikertaramoor). Fire treatment sites were in natural savanna vegetation areas between plantation blocks. Mean annual rainfall is shown for each site, collected from manual rain gauges between 2005/06 and 2008/09.

**Figure 2:**
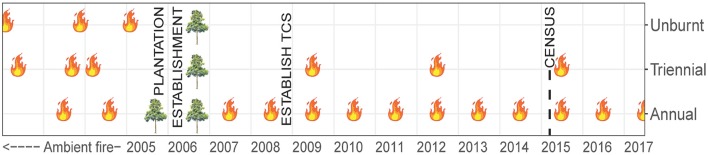
Timeline of fire history at Tiwi Carbon Study (TCS) fire treatment sites. Unburnt: fire-excluded for 10 years, Triennial: burnt every 3 years in the early dry season, Annual: burnt annually in the early dry season. Data used in our study were collected in May 2015, immediately prior to implementation of that year's planned burn program and 6 years after the first experimental burns of the Tiwi Carbon Study in 2009. Prior to plantation establishment in 2005/06 the study sites were subjected to ambient, unquantified, fire regimes.

Data presented in this study were collected within experimental fire areas established as part of the Tiwi Carbon Study. Detailed information about the Tiwi Carbon Study, including study area descriptions can be found in Richards et al. (2012). In brief, the Tiwi Carbon Study consisted of 18 treatment sites at four locations in western Melville Island (Figure 1). Annual early dry season burning (annual), early dry season burning every 3 years (triennial) and fire exclusion (unburnt) treatments were replicated across these locations. There was one replicate of each fire treatment at Imalu, Taracumbi and Pikertaramoor, and three replicates of each fire treatment at Shark Bay (total of six replicates for each fire treatment). All fire treatment sites were located in natural savanna vegetation areas between forestry plantation blocks of *Acacia mangium* Willd. These areas burnt frequently before plantation establishment in 2006 (2005 at Imalu). Since 2001, ~35% of the eucalypt open forests on the Tiwi Islands have burnt every year, 72% of which occurred in the late dry season (Richards et al., 2015). Unburnt fire treatment sites have had fire excluded since plantation establishment. Annual treatment sites were burnt annually since plantation establishment and triennial treatment sites had fire excluded between plantation establishment and 2009, when the first experimental burns of the Tiwi Carbon Study were conducted (Figure 2). After 2009, fire intensities at the annual and triennial fire treatments were determined from scorch heights at each site using equations from Williams et al. (2003, p. 40). Fire intensities averaged 489 [± standard error (se) 96] kw m^−1^ at the annual treatment sites and 2020 (± se 556) kw m^−1^ at triennial treatment sites. Burn coverage (patchiness) averaged 73% (± se 5) in the annual fire treatment and 84 (± se 6)% in the triennial fire treatment (Appendix S1).

### Data collection

At each fire treatment site, nested variable-width transects were established. Ramets of woody species that have the potential to become trees (woody plant with a single dominant trunk and >6 m tall) were counted within each of six size classes. Shrubs, palms and cycads were excluded from the census. Small resprouts (<0.5 m tall) and advanced resprouts (0.5–1.5 m tall) were counted in a 2 × 180 m transect. Saplings (>1.5 m tall but <5 cm diameter at breast height) were counted in a 4 × 180 m transect, and poles (5–10 cm DBH) and canopy trees (subdivided into two classes: 10–20 cm DBH and >20 cm DBH) were counted within a 10 × 180 m transect. The census was undertaken at the end of the wet season, in May 2015 (Figure 2). At the time, triennial fire treatment sites had been unburnt for 3 years and were due to be burnt in the following dry season (June-July 2015). No *A. mangium* plantation wildlings were encountered in the census.

Tree species were classified into eco-taxonomic groups: eucalypts, pantropicals, acacias, and Proteaceae. Eucalypts were any species from the genera *Eucalyptus* and *Corymbia*. Pantropicals included a range of species of pantropical affiliation that are frequent, but sub-dominant components of northern Australian mesic savannas, including species from the families Caesalpiniaceae, Rhamnaceae, Anarcardiaceae, Lecythidaceae, Combretaceae, Malvaceae, and Myrtaceae. Acacias were any species from the genus *Acacia*, and Proteaceae were any species of the family Proteaceae. These classifications are based on the groups defined by Werner (2005) and Prior et al. (2009).

### Data analysis

All analyses were undertaken in the open source software R (R Core Team, 2017). First, we modeled fire treatment effects on tree density in each size class using generalized linear mixed models (GLMMs). The response variable was the count of individuals in a size class with fire treatment as the predictor. The unburnt fire treatment was assigned as the reference class. Diagnostics of initial poisson (log link) model fits indicated overdispersion, which was rectified by re-fitting the models with negative binomial errors with log link (Gelman and Hill, 2009; Appendix S2). A site random effect was included to account for dependency among transects at the same site. We did investigate a random slope model with fire treatment effect varying by site. Model comparison by parametric bootstrap using R package “pbkrtest” suggested no support for the more complex model, as indicated by *p*-values on the likelihood ratio test statistic, therefore we proceeded with the random intercept-only model (Halekoh and Højsgaard, 2014; Appendix S2). Plot size was included as an offset, accounting for the variable width transects so that the response (raw count value) was interpreted as a density and therefore directly comparable between all size classes. Models were fit using the packages “broom” (Robinson, 2015) and “lme4” (Bates et al., 2014).

Next, we modeled fire treatment effects by eco-taxonomic group, using the model format described above. Again, our response variable was count of individuals in a size class, however this time we included fire treatment, eco-taxonomic group and their interaction as predictors. Eucalypts in the unburnt treatment were the reference class. We excluded the canopy size classes from this analysis. Within each fire treatment, the canopy size classes (10–20 cm DBH and >20 cm DBH) were dominated by eucalypts, with many fewer pantropicals and acacias and often no Proteaceae. As such, canopy size class models of the interaction between fire treatment and eco-taxonomic group were untenable due to model convergence issues. Given there is little evidence that mild fire regimes or fire exclusion significantly alter canopy tree composition after 23 years (Russell-Smith et al., 2003), we did not expect to detect change in the canopy after just 9 years of fire exclusion. We therefore focus on the resprout and midstorey (sapling and pole) size classes, which are more likely to be directly impacted by fire in the short-term.

Marginal predictions and 95% confidence intervals of fixed effects from all models described above were obtained via parametric bootstrap using “lme4” (Bates et al., 2014). R code used for all analyses is available in Appendix S2.

## Results

### How does fire frequency affect tree densities within different size classes?

**Figure 3:**
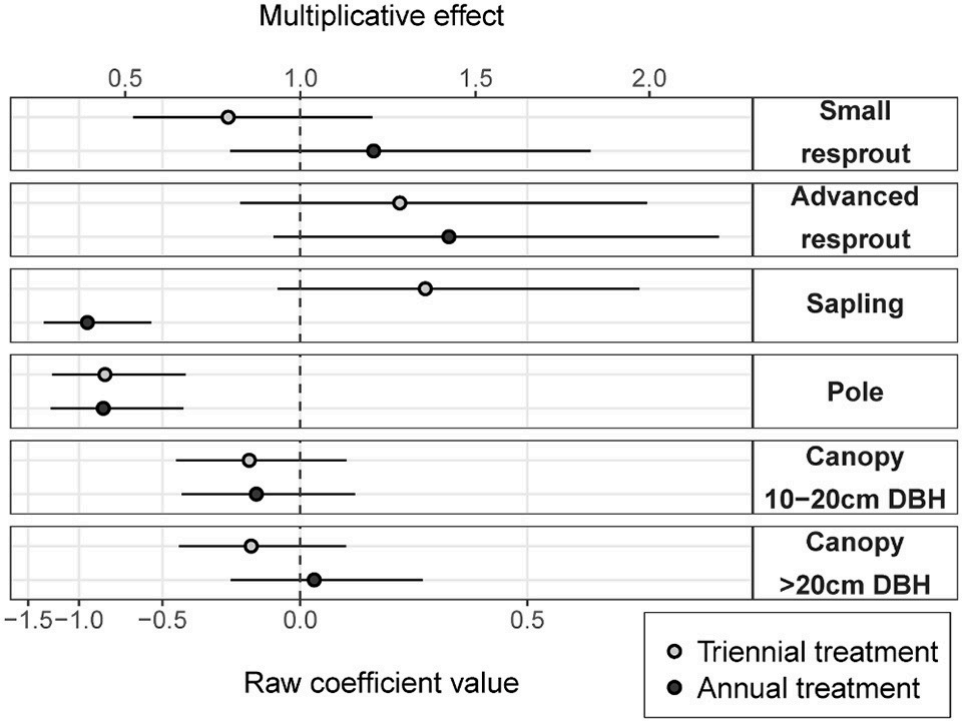
Annual and triennial fire treatment coefficients (raw coefficient values on the log scale) relative to the unburnt reference class (dashed line). Error bars are 95% confidence intervals. The multiplicative effect is the exponentiated coefficient value and indicates relative change in density of each size class in annual and triennial fire treatments compared to unburnt.

**Figure 4:**
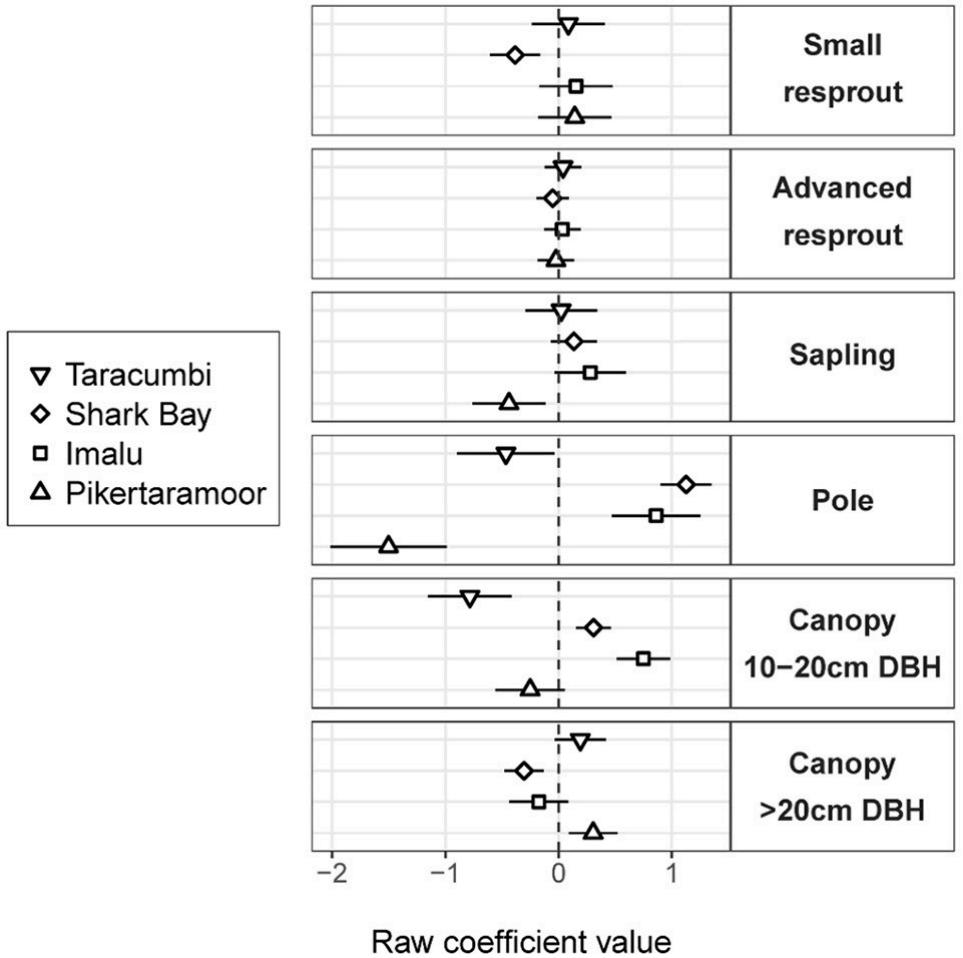
Site intercepts (raw coefficient values on the log scale), which indicate departures of tree density within each size class from the average site (dashed line). Error bars are 95% confidence intervals. Sites are shown in order of decreasing rainfall from top to bottom within each size class.

Fire treatment was an important predictor of tree density in the midstorey size classes (i.e., saplings and poles; Figure 3). In the largest (canopy) and smallest (resprout) size classes there was no detectable effect of fire treatments on tree density. There were site effects on tree densities, which were most pronounced in the pole and canopy size classes (Figure 4). Compared to the average, canopy and pole densities were lowest in the wettest (Taracumbi) and in the driest (Pikertaramoor) sites.

Compared to the unburnt fire treatment, sapling density was on average 1.3 times greater under triennial burning (although the effect was not significantly different from zero), but was (significantly) halved under annual burning (Figure 3). Pole density was (significantly) halved under both triennial and annual burning compared to the unburnt fire treatment. There were no significant differences within any other size class, however, there was a trend toward more small resprouts in the annual fire treatment and more advanced resprouts under both annual and triennial fire treatments compared to the unburnt. The effects of fire treatment on stem densities in the resprout size classes were uncertain, reflecting large unexplained variance. Given small predicted effect sizes with relatively narrow confidence limits in the canopy size classes, we can be confident that fire frequency had had little effect on canopy-tree densities after 6 years of fire treatment (Figure 3).

**Figure 5:**
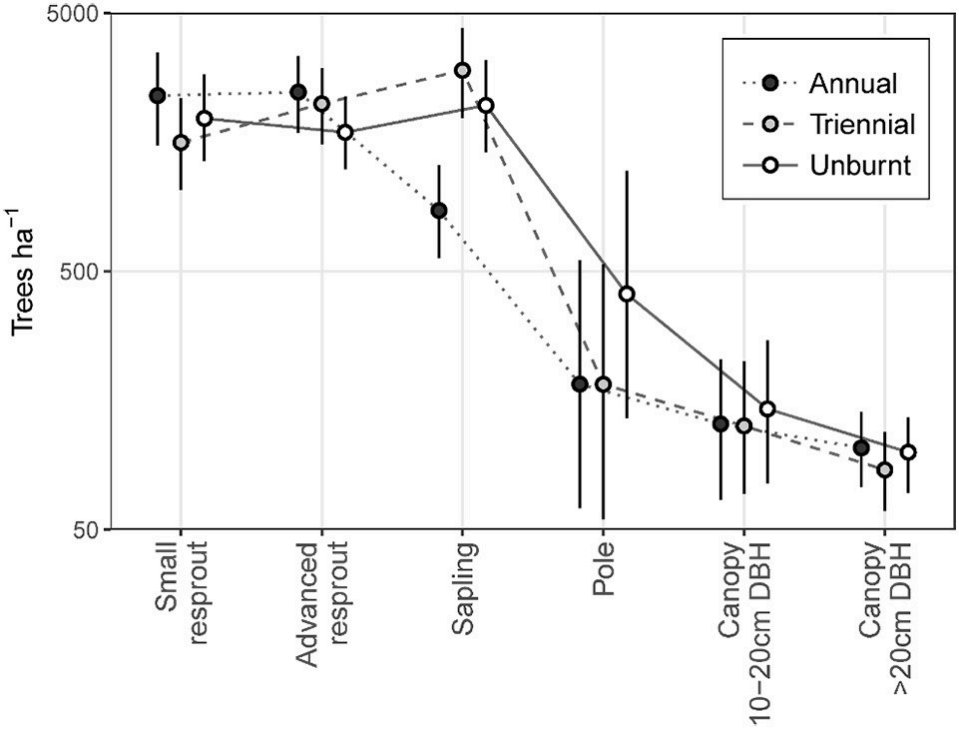
Tree size class distributions in annual, triennial, and unburnt fire treatments. Error bars are 95% confidence intervals.

The annual fire treatment was resprout-dominated, leading to a size class distribution that peaked at the resprout stage then steeply declined toward the canopy (Figure 5). By contrast, triennial and unburnt fire treatment sites were characterized by tree size class distributions that peaked at the sapling stage before steeply declining toward the pole and canopy size classes. Pooling all size classes, mean total tree density did not differ significantly between fire treatments. However, total density was highest in the triennial fire treatment [7,610 (± se 1,162) trees ha^−1^] and lowest in the annual [6,168 (± se 523) trees ha^−1^] fire treatment. Unburnt total tree densities were 7,051 (± se 578) trees ha^−1^.

### How does fire frequency affect tree densities within and between eco-taxonomic groups?

**Figure 6:**
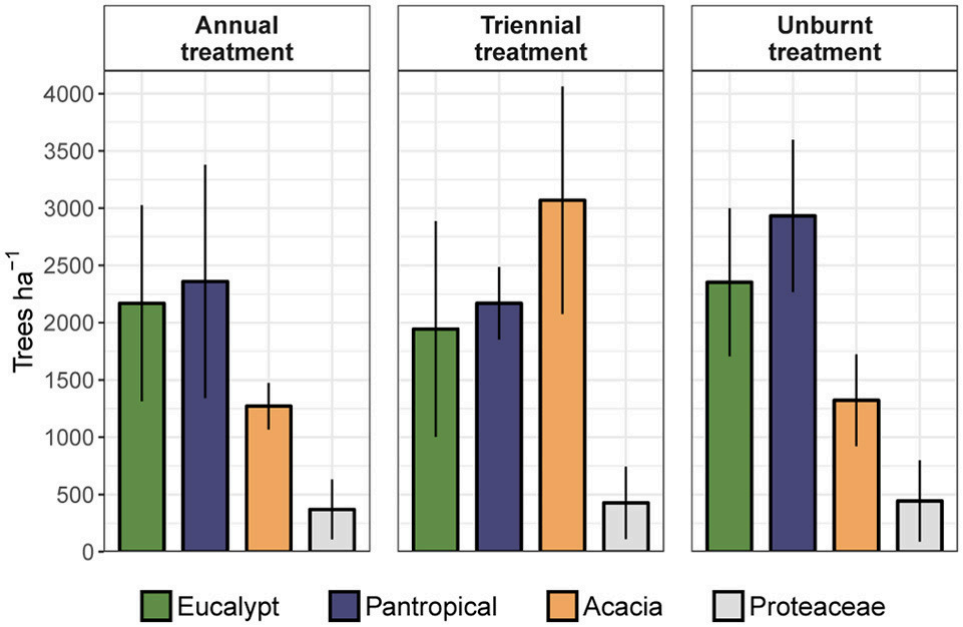
Mean total density (± 95% confidence interval) of trees in each eco-taxonomic group by fire treatment.

**Figure 7:**
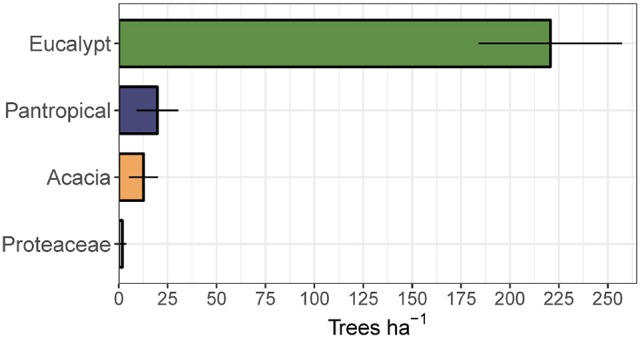
Mean total canopy tree (>10 cm diameter at breast height) density (±95% confidence interval) by eco-taxonomic group, averaged across fire treatments.

To assess the relative contribution of each eco-taxonomic group to the overall tree community, we calculated mean tree density within each eco-taxonomic group and fire treatment (Figure 6). Total densities of pantropicals did not differ from eucalypts under either the annual or triennial fire treatments and were only marginally higher than eucalypts in the unburnt fire treatment. The high tree densities in the triennial fire treatment reported above were predominantly due to a more than doubling of acacia density compared to annual and unburnt fire treatments. Conversely, eucalypt and pantropical densities were at their lowest in the triennial and highest in the unburnt fire treatment. Proteaceae were consistently sub-dominant elements of these savannas and their average density did not vary significantly between fire treatments (Figure 6). Eucalypts dominated the overstorey (Figure 7).

**Figure 8:**
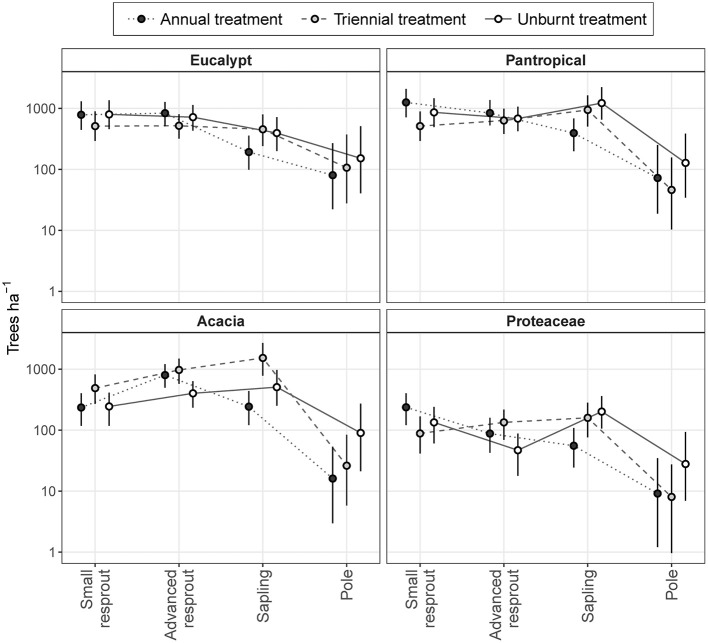
Model predictions of sub-canopy tree size class distributions in annual, triennial, and unburnt fire treatments by eco-taxonomic group. Error bars are 95% confidence intervals.

Across all eco-taxonomic groups, differences in sub-canopy size class distributions between fire treatments were predominantly due to differing sapling densities (Figure 8). Eucalypt sub-canopy size class distributions remained relatively stable across fire treatments, with only a slight depression in sapling density in the annual fire treatment. In contrast, pantropical and Proteaceae sub-canopy size class distributions shifted from consistently decreasing with increasing size in the annual fire treatment to peaking at the sapling stage in the triennial and unburnt fire treatments. Acacia sub-canopy size class distributions were consistently unimodal, but peaked at different size classes: the advanced resprout size in the annual treatment and the sapling size class in the triennial and unburnt fire treatments (Figure 8).

The high total densities of saplings in the triennial and unburnt fire treatments found in our first analysis (Figure 5) were predominantly due to increased acacia and pantropical sapling densities with decreased fire frequency (Figure 8). Proteaceae and eucalypt sapling densities also increased under triennial and unburnt fire treatments relative to the annual fire treatment, but to a lesser extent. Pole densities varied somewhat between fire treatments in each eco-taxonomic group, tending to be highest in the unburnt treatment, but these effects were not significant (Figure 8). Despite eucalypts overwhelmingly dominating the canopy (Figure 7), eucalypt densities usually equaled, and in some size classes were outnumbered by, pantropicals and acacias in sub-canopy size classes across all fire treatments (Figure 8).

## Discussion

Our results from a mid-term fire experiment in a northern Australian mesic savanna demonstrate that saplings are very responsive to differences in fire frequency and that, with at least 3 fire-free years, significant midstorey thickening is possible. These results align with the findings of longer-term savanna fire experiments conducted elsewhere (Russell-Smith et al., 2003; Higgins et al., 2007; Smit et al., 2010), and suggest the presence of a demographic bottleneck limiting recruitment of trees into larger size classes (Prior et al., 2010). The responsiveness of the midstorey to different fire frequencies may result in enhanced carbon sequestration potential at sites subjected to less frequent fires. However, such midstorey thickening also represents a substantially higher fuel load that may lead to higher fire intensities when fire is re-introduced (Appendix S1). Managers should therefore be wary of the potential for high carbon loss and increased greenhouse gas emissions in fire years with less frequent fire regimes due to increased fire severity (Murphy and Russell-Smith, 2010). There was a trend toward decreased resprout densities and increased pole densities with less frequent fire, as per our expectations, but these effects were less pronounced. Eco-taxonomic groups did differ in their responsiveness to reductions in fire frequency, supporting assertions that different eco-taxonomic groups have different capacities to escape from the frequent fire trap (Bond et al., 2012). In particular, eucalypt densities did not substantially differ in any size class between fire treatments compared to the other eco-taxonomic groups, indicating that fire may not be the main factor limiting eucalypt tree densities (Murphy et al., 2015).

There was often wide confidence limits around our model estimates, reflecting relatively large unexplained variance among sites. These site differences were likely due to variability in fire intensity and burn patchiness (Hoffmann, 2000; Appendix S1). Site-based differences in burn characteristics are largely self-perpetuating as hotter fires may favor grasses and more complete burn coverage, which in turn affects the size at which stems become resilient to topkill (Scholes and Archer, 1997; Higgins et al., 2000). Rainfall can also influence woody vegetation structure (Sankaran et al., 2005), however here we found pole and canopy tree variation between sites was not correlated with mean annual rainfall, substantiating the findings of others that soil factors such as texture (Williams et al., 1996) and fertility (Lehmann et al., 2011) may play a critical role in moderating tree cover in mesic savannas. Whether, given more time, fire frequency effects on smaller size classes would amount to canopy tree density differences between fire regimes cannot be determined from our study, but evidence from long-term fire experiments elsewhere in Australia suggests this may not be the case, with effects confined to the midstorey (Russell-Smith et al., 2003; Woinarski et al., 2004).

The negative relationship between sapling density and frequent fire found here is well-documented elsewhere and reflects high susceptibility of saplings to topkill (Fensham, 1990; Bond and Van Wilgen, 1996; Peterson and Reich, 2001). However, under the low fire intensities of this study (Appendix S1) and considering results from other studies that show trees subjected to low intensity fire can resist topkill at 5 cm diameter (Lawes et al., 2011a) we expect the pole size class should largely be impervious to topkill by fire. Trends in pole densities are therefore unlikely to be due to suppression by topkill of this size class, but rather due to topkill of smaller individuals limiting growth to pole size. For example, slightly higher pole densities in the unburnt, but not the triennial fire treatment provided evidence that most saplings were topkilled by fire in the triennial treatment, preventing their growth into the pole size class. This indicates a rolling cycle of topkill, resprouting and growth through to sapling size within 3 years, testament to the high productivity of the Tiwi Islands compared to mainland Australian savannas (Richards et al., 2012). On the mainland, increased sapling densities were detected after 5 years of fire exclusion (Russell-Smith et al., 2003).

Consistent with findings from other long-term northern Australian fire experiments (Andersen et al., 2003; Russell-Smith et al., 2003; Woinarski et al., 2004), we found that eucalypt size class distributions remained relatively stable across fire treatments, but there were shifts toward unimodal distributions (due to increased sapling density) with decreasing fire frequency in the other eco-taxonomic groups. Such results are consistent with hypotheses of Australian savanna dynamics that suggest eucalypts are not limited by fire in reaching the canopy while pantropicals are (Bond et al., 2012; Murphy et al., 2015). This has also been reflected in detection of escape for eucalypts, but mixed ability to detect escape in some pantropical species, particularly under more intense fire regimes (Bond et al., 2012; Freeman et al., 2017). Yet the predominance of pantropicals in sub-canopy size classes does belie the fire-sensitive label bestowed on pantropicals within Australian savanna literature, which has led to the pervasive understanding that these species are not successful under frequent fire (Box 1).

Box 1 **A selection of quotes highlighting the bias in current australian savanna literature toward a perception that non-eucalypt and pantropical tree species are sensitive to fire and therefore less successful than eucalypts. these assertions assume that all savanna tree species are juvenile as resprouts and therefore have the same imperative to escape from fire suppression in the resprout size class to reach maturity in the canopy.**

“…the success of the eucalypts in Australian savannas is related to their fire tolerance relative to other eco-taxonomic groups.” (Prior et al., 2010, p. 602)

“…the deciduous element of the tree flora is more fire-sensitive than the evergreen element.” (Lawes et al., 2011a, p. 3)

“Non-eucalypts are very common as suppressed juvenile plants over large areas but are rare and patchy components of the large-tree layer.” (Bond et al., 2012, p. 679)

“…Myrtaceous species account for 60–80% of standing biomass in this region, perhaps because they are more fire tolerant than non-Myrtaceous species.” (Prior et al., 2004, p.313)

“The concept that eucalypts are less sensitive to fire than non-eucalypts suggests that frequent fires may reduce non-eucalypt basal area relative to eucalypt basal area.” (Lawes et al., 2011b, p. 166)

“It is only the fire-sensitive non-eucalypts of the midstorey – mostly broadleaved, deciduous species—that respond very positively to reductions in fire frequency and intensity.” (Murphy et al., 2015, p. 9)

“Most tree diversity in regional savannas comprises non-eucalypt, typically smaller-statured taxa which are relatively susceptible to severe fires.” (Russell-Smithet et al, 2012, p. 1308)

“…fire-sensitive pantropics were particularly disadvantaged.” (Werner, 2005, p. 625)

“…non-eucalypts were favoured by long fire free intervals.” (Bond et al., 2012, p.681)

“Only by growing through the sapling stage and attaining sufficient height or bark thickness are savanna trees able to escape the ‘fire trap’ and reach maturity” (Murphy and Bowman, 2012, p. 4)

“…without fire, the non-eucalypt, fire-sensitive, tree species become a more important part of the woody component of these savannas” (Werner and Prior, 2013, p.88)

**Figure 9:**
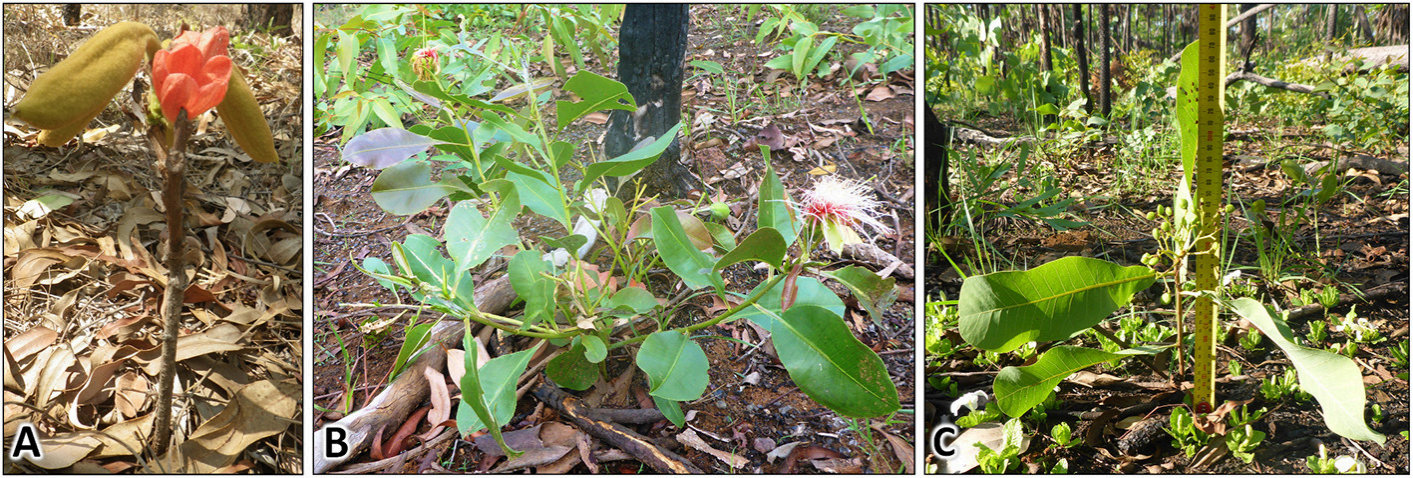
Species in the pantropical eco-taxonomic group that are capable of fruiting and flowering at very small height within the flame zone and within 1 year of burning. **(A)** *Brachychiton megaphyllus* (Malvaceae), **(B)** *Planchonia careya* (Lecythidaceae), **(C)** *Buchanania obovata* (Anarcardiaceae).

We propose that the lack of pantropicals in the canopy indicates distinct strategies between eucalypts and pantropicals that, to our knowledge, have not been formally recognized. The primary author (M.E. Freeman) recorded informal observations of flowering and fruiting for the 11 most common tree species encountered in our census in a separate growth study of 1,250 healthy resprout and sapling-sized trees (<5 cm diameter) at the same fire experiment sites as this study (unburnt and annual fire treatment sites only, Freeman, 2017; Table 1). She observed that several pantropicals flower and fruit within the flame zone as low as 5 cm in height and within 1 year of resprouting after fire-induced topkill (Table 1, Figure 9). Eucalypts purportedly do not become sexually mature until ~7–10 cm diameter (i.e., pole-sized individuals) (N. Cuff and P. McCullough pers. comm.). This suggests that eucalypts are predominantly “gullivers” [i.e., suppressed juveniles, *sensu* Bond and Van Wilgen (1996)], but many pantropicals are not.

**Table 1:** Informal observations of flowering (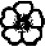) and fruiting (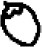) from 1,250 resprout and sapling sized trees (<5 cm diameter at 50 cm above ground) measured in a growth study of 11 common species at the same annual (A) and unburnt (U) fire experiment sites as censused in this study (Freeman 2017).

**Species**	**Month observed**					**Observations of reproductive height (m)**		**N**		**Max. ht (m)**
	**November**		**August**	**May**				
	**A**	**U**	**A**	**A**	**U**	**A**	**U**	**A**	**U**	
**EUCALYPTS**										
*Corymbia nesophila* (Blakeley) K.D.Hill & L.A.S.Johnson	–	–	–	–	–	–	–	76	85	30
*Eucalyptus miniata* A.Cunn. ex Schauer	–	–	–	–	–	–	–	87	60	30
*Eucalyptus tetrodonta* F.Muell.	–	–	–	–	–	–	–	80	87	30
**PANTROPICALS**										
*Alphitonia excelsa* (A.Cunn. ex Fenzl) Benth	–	–	–	–	–	4.2	–	73	49	15
*Brachychiton megaphyllus* Guymer	–	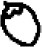	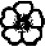	–	–	0.9, 1.4, 1.6	0.6, 1.0, 2.1, 2.7, 3.0	21	31	8
*Buchanania obovata* Engl.	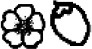	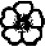	–	–	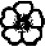	0.2, 0.2, 0.3, 0.4, 0.5, 3.8	0.6, 0.8, 1.2, 1.5, 2.0, 2.1, 2.1, 2.4, 3.1	40	48	10
*Erythrophleum chlorostachys* (F.Muell.) Baill.	–	–	–	–	–	–	–	45	52	18
*Planchonia careya* (F.Muell.) R.Knuth	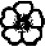	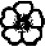	–	–	–	0.05, 0.3, 0.4, 0.4, 0.5	0.1, 0.2, 0.3, 0.4, 1.0, 1.2, 1.2, 1.4	74	77	15
*Terminalia ferdinandiana* Exell	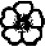	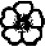	–	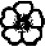	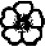	0.3, 0.4, 0.5, 0.9, 3.4	0.7, 1.0, 1.7, 2.0, 2.1	42	33	14
**ACACIA**										
*Acacia latescens* Benth	–	–	–	–	–	6.5	–	36	30	10
**PROTEACEAE**										
*Grevillea heliosperma* R.Br.	–	–	–	–	–	–	4.3	75	49	12

*Fecundity observations were recorded as presence or absence of reproductive material between November 2014 and May 2016. A “–”indicates that no fruits or flowers were observed on these species over the observation period. N is the total number of individuals surveyed. Max. ht. (m) is the maximum potential height of the species according to Brock and Dunlop (2007)*.

Gullivers persist as juvenile resprouts and rely on escape from the fire trap (repeated topkill) to reach sexual maturity (Bond and Van Wilgen, 1996; Higgins et al., 2012). Two of the dominant eucalypts in this study, *E. miniata* and *C. nesophila*, do not reproduce clonally and therefore risk population extinction if they do not escape the fire trap and reproduce sexually. Granted, current high numbers of established gullivers awaiting a window of opportunity to escape, coupled with the relative longevity of canopy trees means population extinction is highly unlikely over foreseeable timeframes (Fensham and Bowman, 1992). But, this potential extinction risk is a defining feature of these species' growth strategies and makes escape from the fire trap an imperative. *E. tetrodonta* can produce rhizomes, however such a strategy without input of new genetic material via seedlings similarly poses a risk to population viability in the long-term. Eucalypts are obligate trees—they must advance to larger size classes if they are to sexually reproduce.

In contrast, although many Australian pantropicals have been described as fire-sensitive trees that commonly exist as suppressed juveniles (Box 1), we propose that many are actually facultative trees: successful shrubs which, under the right conditions, have the ability to become trees. They persist in high numbers as resprouts under frequent fire and direct energy to sexual reproduction within the flame zone (Figure 9). This suggests a growth strategy not defined by escaping from frequent fire but rather aimed at persisting and reproducing within it. We acknowledge that fire-stimulated flowering exists and that fire frequency would prevent most seedlings from establishing in the inter-fire period (Hoffmann, 2000; Lamont and Downes, 2011). Nevertheless, we make the point that there is a critical distinction between facultative trees and obligate trees that will define population responses to disturbance and that demonstrate different ways to succeed.

Zizka et al. (2014) explicitly defined a spectrum from “shrub” to “shrub-sometimes-small-tree” to “tree” in African savannas, which should be formally tested in Australia. We suggest this classification would prove invaluable to the way we conceptualize tree dynamics and perceive woody plant success in Australian savannas. Interactions between fire and reproduction for different woody species have been acknowledged as important drivers of savanna dynamics elsewhere (Hoffmann, 1998; Gardner, 2006; Zizka et al., 2014; Pilon and Durigan, 2017), but have not yet been adequately explored in Australia. To truly determine resprouting woody species success and predict advance of resprouts to the canopy, fecundity data and information on minimum reproductive heights for a range of species and environmental conditions is required. Such information is critical to inferring the sustainability of management regimes and predicting the long-term impacts of fire management on savanna structure and composition.

## Conclusions

This study showed significant effects of fire frequency on savanna structure and the relative dominance of eco-taxonomic groups within size classes in the wettest savannas of monsoonal northern Australia. These results concur with findings of previous studies in the region (Andersen et al., 2003; Russell-Smith et al., 2003; Woinarski et al., 2004). This congruence is quite remarkable given that variable rainfall, soil and fire characteristics across sites could foreseeably lead to quite different outcomes. What has not been identified before, however, are the fundamental differences in strategies of Australian savanna trees. Frequent fire limits pantropical, acacia and Proteaceae species from advancing to larger size, whereas eucalypts remain relatively unresponsive to fire regime (Murphy et al., 2015). The scarcity of non-eucalypts in the canopy has fueled arguments that these groups are more fire-sensitive and less successful than eucalypts. A common understanding is that pantropicals are favored by long fire-free intervals (Bond et al., 2012). Our results concur with such statements if success is defined as the ability to reach maximum height potential. However, we have shown that pantropicals equal or outnumber eucalypts in sub-canopy size classes across different fire treatments and may be sexually reproductive while in the flame zone. Greater understanding of reproductive strategies, which should affect a species' imperative to escape the fire trap, will be critical to better predicting tree dynamics under different management and climate scenarios.

## Data availability statement

The raw data supporting the conclusions of this manuscript will be made available by the authors, without undue reservation, to any qualified researcher.

## Author contributions

MF, BM, AR, and GC conceived the ideas; MF and BM collected the data; MF: analyzed the data with input and review from BM, AR and PV; MF wrote the paper with input and review from BM, AR, PV, and GC; MF, BM, AR, PV, and GC approved the final version.

### Conflict of interest statement

BM is a topic editor for the research topic Proceedings of the symposium Wildfire Ecology and Life Evolution: From Ancient Time to Present of the International Congress of Ecology (INTECOL 2017 Beijing) to which this manuscript has been submitted. The other authors declare that the research was conducted in the absence of any commercial or financial relationships that could be construed as a potential conflict of interest.

## Supplementary material

Appendix S1 Site information. Table summarizing average rainfall and fire metrics for each Tiwi Carbon Study transect.

Appendix S2 R script and model output. R code used for data preparation, model building, overdispersion tests, model comparison and prediction via parametric bootstrap. Output of overdispersion tests and model comparisons are embedded in the document.
