# Supplementary material for: Facultative and Obligate Trees in a Mesic Savanna: Fire Effects on Savanna Structure Imply Contrasting Strategies of Eco-Taxonomic Groups
Source: Front Plant Sci. 2018 May 18;9:644. doi: 10.3389/fpls.2018.00644 (PMC5968114; doi:10.3389/fpls.2018.00644)
Supplement: Appendix S1 — Site information. Table summarizing average rainfall and fire metrics for each Tiwi Carbon Study transect. [file Data_Sheet_1.docx]

Supplementary Material

Facultative and obligate trees in a mesic savanna: Fire effects on savanna structure imply contrasting strategies of eco-taxonomic groups

Michelle E. Freeman*, Brett P. Murphy, Anna E. Richards, Peter A. Vesk, Garry D. Cook

*** Correspondence:** Corresponding Author: [freemanm@student.unimelb.edu.au](mailto:freemanm@student.unimelb.edu.au)

# Appendix S1 - Tiwi Carbon Study site information

Fire metrics were averages of annual data collected between 2009 – 2015 as described by Richards et al. (2012). Fire intensity was calculated from scorch height using equations in Williams et al. (2003).

| **Site** | **Rainfall (mm)** | **Plot** | **Fire treatment** | **Proportion burnt** | **Fire intensity (kw m^-1^)** |
| --- | --- | --- | --- | --- | --- |
| Imalu | 1194 | 1 | Annual | 87% | 964 |
|  |  | 2 | Triennial | 100% | 6744 |
|  |  | 3 | Unburnt | *NA* | *NA* |
| Pikertaramoor | 1252 | 4 | Annual | 94% | 527 |
|  |  | 5 | Triennial | 99% | 1927 |
|  |  | 6 | Unburnt | *NA* | *NA* |
| Shark Bay | 1369 | 7 | Annual | 45% | 185 |
|  |  | 8 | Triennial | 83% | 371 |
|  |  | 9 | Unburnt | *NA* | *NA* |
|  |  | 10 | Annual | 50% | 124 |
|  |  | 11 | Triennial | 48% | 251 |
|  |  | 12 | Unburnt | *NA* | *NA* |
|  |  | 13 | Annual | 72% | 393 |
|  |  | 14 | Triennial | 77% | 1682 |
|  |  | 15 | Unburnt | *NA* | *NA* |
| Taracumbi | 1544 | 16 | Annual | 91% | 742 |
|  |  | 17 | Triennial | 98% | 1148 |
|  |  | 18 | Unburnt | *NA* | *NA* |

Richards, A. E., A. N. Andersen, J. Schatz, R. Eager, T. Z. Dawes, K. Hadden, K. Scheepers, and M. Van Der Geest. 2012. Savanna burning, greenhouse gas emissions and indigenous livelihoods: Introducing the Tiwi Carbon Study. Austral Ecology **37**:712-723.

Williams, R. J., A. M. Gill, and P. H. Moore. 2003. Fire behaviour. Pages 33-46 *in* A. N. Andersen, G. D. Cook, and R. J. Williams, editors. Fire in tropical savannas. Springer.

# Appendix S2 - R Script

### Load packages

library(plyr)
library(reshape2)

library(dplyr)
library(broom)
library(lme4)
library(ggplot2)
library(pbkrtest)

### Data description

There are:

Three fire treatments: - Annual early dry season burning - Triennial early dry season burning - Fire exclusion (Unburnt)

Four eco-taxonomic groups: - Eucalypts - Pantropicals - Acacias - Proteaceae

Six size classes: - <0.5 m tall = small resprout - 0.5-1.5 m tall = advanced resprout - >1.5 m tall but < 5 cm diameter at breast height (DBH) = sapling - 5 to 10 cm DBH = pole - 10 to 20 cm DBH = canopy - >20 cm DBH = canopy

Four sites and 18 plots in total (six plots per fire treatment): - Imalu: one replicate of each fire treatment - Shark Bay: three replicates of each fire treatment - Taracumbi: one replicate of each fire treatment - Pickertaramoor: one replicate of each fire treatment

Transects within a plot were variable width depending on size class:

- small resprout = 2 m by 180 m (360 m^2)
- advanced resprout = 2 m by 180 m (360 m^2)
- sapling = 4 m by 180 m (720 m^2)
- pole = 10 m by 180 m (720 m^2)
- canopy = 10 m by 180 m (1800 m^2)

### Load data

CountData has the following columns: - FireTrt = fire treatment - EcoGrp = eco-taxonomic group - SizeClass = size class - Site = site - Plot = arbitrary plot number from 1 to 18 - PlotSize = size of transect in m^2 - Count = number of individuals

CountData <- read.csv("CountData.csv", header = T, sep = ",")

CountData <-
 CountData %>%
 #Ensure factors are in a good order:
 mutate(FireTrt = factor(FireTrt, levels = c("Unburnt", "Annual", "Triennial")),
 SizeClass = factor(SizeClass, levels = c("<0.5m", "0.5-1.5m", ">1.5m", "5-10DBH","10-20DBH", ">20DBH")),
 EcoGrp = factor(EcoGrp, levels = c("Eucalypt", "Pantropical", "Acacia", "Proteaceae")),
 #Convert counts to trees per hectare:
 Ha_multiplier = 10000 / PlotSize,
 TreesHa = Count * Ha_multiplier)

## Q1 : Does fire frequency affect tree size distributions of mesic eucalypt savannas?

Here we consider the effect of fire treatment on abundance of individuals within each size class, regardless of eco-taxonomic group

### Prepare data for Q1

DataQ1 <-
 CountData %>%
 #Sum the count of individuals within each FT, SC and Plot. Include Site and PlotSize and Ha_multiplier as grouping variables so dplyr doesn't drop these from the dataframe
 group_by(FireTrt, SizeClass, Plot, Site, PlotSize, Ha_multiplier) %>%
 summarise(Count = sum(Count)) %>%
 ungroup %>%
 #Recalculate per hectare values
 mutate(TreesHa = Count * Ha_multiplier,
 #Set reference value:
 FireTrt = relevel(FireTrt, ref = "Unburnt"))

### Fit poisson model

Fire1 <-
 DataQ1 %>%

#fit each size class as a separate model
 group_by(SizeClass) %>%

 do(model = glmer(Count ~ FireTrt + (1|Site),
 data = . ,
 family = poisson(link = "log"),
 offset = log(PlotSize),
 control = glmerControl(optimizer = "bobyqa",
 optCtrl = list(maxfun = 100000))))

### Test for overdispersion in poisson fit

Overdispersion tests were done using the overdispersion function from <http://glmm.wikidot.com/faq>

Overdispersion test function:

#define overdispersion function

overdisp_fun <- function(model) {
 ##number of variance parameters in an n-by-n variance-covariance matrix
 vpars <- function(m) {
 nrow(m) * (nrow(m) + 1) / 2
 }
 model.df <- sum(sapply(VarCorr(model), vpars)) + length(fixef(model))
 rdf <- nrow(model.frame(model)) - model.df
 rp <- residuals(model, type = "pearson")
 Pearson.chisq <- sum(rp^2)
 prat <- Pearson.chisq / rdf
 pval <- pchisq(Pearson.chisq, df = rdf, lower.tail = FALSE)
 c(chisq = Pearson.chisq, ratio = prat, rdf = rdf, p = pval)
}

The test indicates overdispersion in the sub-canopy size classes (p values <0.05):

ODtest1 <- list()

#for each size class model (1-6):
for (i in 1:6){

#name output
 name <- paste(Fire1$SizeClass[[i]])

#run overdispersion function
 ODtest1[[name]] <- overdisp_fun(Fire1$model[[i]])
}
ODtest1 output:

## $`<0.5m`
## chisq ratio rdf p
## 1.309759e+02 9.355421e+00 1.400000e+01 4.361189e-21
##
## $`0.5-1.5m`
## chisq ratio rdf p
## 1.278553e+02 9.132521e+00 1.400000e+01 1.800656e-20
##
## $`>1.5m`
## chisq ratio rdf p
## 2.146084e+02 1.532917e+01 1.400000e+01 5.616848e-38
##
## $`5-10DBH`
## chisq ratio rdf p
## 1.066557e+02 7.618267e+00 1.400000e+01 2.483817e-16
##
## $`10-20DBH`
## chisq ratio rdf p
## 23.43336216 1.67381158 14.00000000 0.05357347
##
## $`>20DBH`
## chisq ratio rdf p
## 9.7761491 0.6982964 14.0000000 0.7783595

We therefore re-fit models as negative binomial

### Fit negative binomial model

Fire2 <-
 DataQ1 %>%

#fit each size class as a separate model
 group_by(SizeClass) %>%

 do(model = glmer.nb(Count ~ FireTrt + (1|Site) + offset(log(PlotSize)),
 data = . ,
 control = glmerControl(optimizer = "bobyqa",
 optCtrl = list(maxfun = 1000000))))

### Test for overdispersion in negative binomial

Overdispersion tests suggest overdispersion has been rectified by using negative binomial (non-significant p values):

ODtest2 <- list()

#for each size class model (1-6):
for (i in 1:6){
 name <- paste(Fire2$SizeClass[[i]])
 ODtest2[[name]] <- overdisp_fun(Fire2$model[[i]])
}
ODtest2

## $`<0.5m`
## chisq ratio rdf p
## 15.8044942 1.1288924 14.0000000 0.3254589
##
## $`0.5-1.5m`
## chisq ratio rdf p
## 15.0360441 1.0740031 14.0000000 0.3756952
##
## $`>1.5m`
## chisq ratio rdf p
## 13.9765404 0.9983243 14.0000000 0.4514603
##
## $`5-10DBH`
## chisq ratio rdf p
## 13.8427320 0.9887666 14.0000000 0.4614923
##
## $`10-20DBH`
## chisq ratio rdf p
## 13.4465422 0.9604673 14.0000000 0.4917027
##
## $`>20DBH`
## chisq ratio rdf p
## 9.7760844 0.6982917 14.0000000 0.7783642

### Fit random slope model

It is possible that the effect of fire varies by Site, therefore we also fit a random slope model

Fire3 <-
 DataQ1 %>%

#fit each size class as a separate model
 group_by(SizeClass) %>%

 do(model = glmer.nb(Count ~ FireTrt + (FireTrt|Site) + offset(log(PlotSize)),
 data = . ,
 control = glmerControl(optimizer = "bobyqa",
 optCtrl = list(maxfun = 1000000))))

### Compare models

We use parametric bootstrapping to compare our random intercept and random intercept and slope models (see: <http://glmm.wikidot.com/faq>)

PBtest1 <- list()

#for each size class model (1-6):
for (i in 1:6) {
 name <- paste(Fire2$SizeClass[[i]])

#do the model comparison
 PBtest1[[name]] <- PBmodcomp(Fire3$model[[i]], Fire2$model[[i]], nsim = 1000, seed = 84)
}

PBtest1 output:

## $`<0.5m`
## Parametric bootstrap test; time: 585.44 sec; samples: 1000 extremes: 271;
## Requested samples: 1000 Used samples: 935 Extremes: 271
## large : Count ~ FireTrt + (FireTrt | Site) + offset(log(PlotSize))
## small : Count ~ FireTrt + (1 | Site) + offset(log(PlotSize))
## stat df p.value
## LRT 2.9305 5 0.7107
## PBtest 2.9305 0.2906
##
## $`0.5-1.5m`
## Parametric bootstrap test; time: 557.30 sec; samples: 1000 extremes: 636;
## Requested samples: 1000 Used samples: 926 Extremes: 636
## large : Count ~ FireTrt + (FireTrt | Site) + offset(log(PlotSize))
## small : Count ~ FireTrt + (1 | Site) + offset(log(PlotSize))
## stat df p.value
## LRT 1.2989 5 0.9350
## PBtest 1.2989 0.6872
##
## $`>1.5m`
## Parametric bootstrap test; time: 609.44 sec; samples: 1000 extremes: 512;
## Requested samples: 1000 Used samples: 897 Extremes: 512
## large : Count ~ FireTrt + (FireTrt | Site) + offset(log(PlotSize))
## small : Count ~ FireTrt + (1 | Site) + offset(log(PlotSize))
## stat df p.value
## LRT 2.716 5 0.7437
## PBtest 2.716 0.5713
##
## $`5-10DBH`
## Parametric bootstrap test; time: 599.15 sec; samples: 1000 extremes: 117;
## Requested samples: 1000 Used samples: 909 Extremes: 117
## large : Count ~ FireTrt + (FireTrt | Site) + offset(log(PlotSize))
## small : Count ~ FireTrt + (1 | Site) + offset(log(PlotSize))
## stat df p.value
## LRT 6.4661 5 0.2635
## PBtest 6.4661 0.1297
##
## $`10-20DBH`
## Parametric bootstrap test; time: 375.26 sec; samples: 1000 extremes: 826;
## Requested samples: 1000 Used samples: 868 Extremes: 826
## large : Count ~ FireTrt + (FireTrt | Site) + offset(log(PlotSize))
## small : Count ~ FireTrt + (1 | Site) + offset(log(PlotSize))
## stat df p.value
## LRT 0.5941 5 0.9883
## PBtest 0.5941 0.9517
##
## $`>20DBH`
## Parametric bootstrap test; time: 489.95 sec; samples: 1000 extremes: 291;
## Requested samples: 1000 Used samples: 984 Extremes: 291
## large : Count ~ FireTrt + (FireTrt | Site) + offset(log(PlotSize))
## small : Count ~ FireTrt + (1 | Site) + offset(log(PlotSize))
## stat df p.value
## LRT 1.952 5 0.8557
## PBtest 1.952 0.2964

There is no evidence that the random slope model is preferred over the random intercept model. We use the model Fire2 for subsequent predictions.

### Get predictions by parametric bootstrap

Bootstrap functions are modified from code published at these sites: <https://cran.r-project.org/web/packages/merTools/vignettes/Using_predictInterval.html> <https://stat.ethz.ch/pipermail/r-sig-mixed-models/2014q3/022521.html>

Bootstrap function:

#define function that generates marginal predictions
predictFun <- function(x) {
 predict(x, newdata = ND, re.form = NA, type = "response")
}

#define function that summarises bootstrap into median and 95% CI
sumBoot <- function(merBoot) {
 return(
 ND %>% bind_cols(
 data.frame(estimate = apply(merBoot$t, 2, function(x) as.numeric(quantile(x, probs = .5, na.rm = TRUE))),
 conf.low = apply(merBoot$t, 2, function(x) as.numeric(quantile(x, probs = .025, na.rm = TRUE))),
 conf.high = apply(merBoot$t, 2, function(x) as.numeric(quantile(x, probs = .975, na.rm = TRUE))))
 )
 )
}

Because we have different plot sizes, we have to specify new data differently for each model.

First predict the small and advanced resprout size classes:

#Define new data

ND <- data.frame(expand.grid(FireTrt = levels(DataQ1$FireTrt),
 PlotSize = 360))

BootF2 <- NULL

#Bootstrap small and advance resprouts
for(i in 1:2) {
 Boot1 <- bootMer(Fire2$model[[i]], predictFun, nsim = 1000, use.u = FALSE, seed = 84)

 BootF2 <-
 #bind each model bootstrap outcome together
 bind_rows(BootF2,
 sumBoot(Boot1) %>%
 #create a column labelling the model
 mutate(Model = i))
}

Then the saplings:

#Define new data

ND <- data.frame(expand.grid(FireTrt = levels(DataQ1$FireTrt),
 PlotSize = 720))

#Bootstrap sapling size class
Boot2 <- bootMer(Fire2$model[[3]], predictFun, nsim = 1000, use.u = FALSE, seed = 84)

BootF2 <-
 #bind each model bootstrap outcome together
 bind_rows(BootF2,
 sumBoot(Boot2) %>%
 #create a column labelling the model
 mutate(Model = 3))

Then the pole and canopy size classes:

#Define new data

ND <- data.frame(expand.grid(FireTrt = levels(DataQ1$FireTrt),
 PlotSize = 1800))

#Bootstrap pole and canopy size classes
for(i in 4:6) {
 Boot3 <- bootMer(Fire2$model[[i]], predictFun, nsim = 1000, use.u = FALSE, seed = 84)

 BootF2 <-
 #bind each model bootstrap outcome together
 bind_rows(BootF2,
 sumBoot(Boot3) %>%
 #create a column labelling the model
 mutate(Model = i))
}

BootF2 <-
 BootF2 %>%

#add SizeClass labels by joining the model summary columns
 inner_join(Fire2 %>%
 rownames_to_column(var = "Model") %>%
 mutate(Model = as.numeric(Model)) %>%
 dplyr::select(-model))

### Site effects summary

The random effects values below give the Site-level errors = the "position" of each Site intercept in relation to the average Site

We use a function written by Ben Bolker to extract random effect estimates and confidence intervals sourced from: <https://stackoverflow.com/questions/34344599/a-caterpillar-plot-of-just-the-significant-random-effects-from-a-mixed-effects>

augment.ranef.mer <- function(x,
 ci.level = 0.95,
 reorder = TRUE,
 order.var = 1) {
 tmpf <- function(z) {
 if (is.character(order.var) && !order.var %in% names(z)) {
 order.var <- 1
 warning("order.var not found, resetting to 1")
 }
 ## would use plyr::name_rows, but want levels first
 zz <- data.frame(level = rownames(z), z, check.names=FALSE)
 if (reorder) {
 ## if numeric order var, add 1 to account for level column
 ov <- if (is.numeric(order.var)) order.var + 1 else order.var
 zz$level <- reorder(zz$level, zz[,order.var + 1], FUN = identity)
 }
 ## Q-Q values, for each column separately
 qq <- c(apply(z, 2, function(y) {
 qnorm(ppoints(nrow(z)))[order(order(y))]
 }))
 rownames(zz) <- NULL
 pv <- attr(z, "postVar")
 cols <- 1:(dim(pv)[1])
 se <- unlist(lapply(cols, function(i) sqrt(pv[i, i, ])))
 ## n.b.: depends on explicit column-major ordering of se/melt
 zzz <- cbind(melt(zz, id.vars = "level", value.name = "estimate"),
 qq = qq, std.error = se)
 ## reorder columns:
 subset(zzz, select = c(variable, level, estimate, qq, std.error))
 }
 dd <- ldply(x, tmpf, .id = "grp")
 ci.val <- -qnorm((1 - ci.level)/2)
 transform(dd,
 p = 2 * pnorm(-abs(estimate/std.error)), ## 2-tailed p-val
 lb = estimate - ci.val * std.error,
 ub = estimate + ci.val * std.error)
}

Get random effects estimates:

SiteRanef <- NULL

#for each size class model (1-6):
for(i in 1:6) {
 SiteRanef <- bind_rows(SiteRanef,
 #extract random effect estimates using augment function
 augment.ranef.mer(ranef(Fire2$model[[i]], condVar = TRUE)) %>%

#create SizeClass column
 mutate(SizeClass = paste(Fire2$SizeClass[[i]])))
}

## Q2 : Does this effect differ between eco-taxonomic groups?

Here we consider the effect of fire treatment on abundance of individuals within the sub-canopy size classes by eco-taxonomic group. The canopy size classes are dominated by eucalypts, with very little or sometimes no representation of other eco-taxonomic groups, therefore models of the 2 canopy size classes are not fit here.

### Prepare data

DataQ2 <-
 CountData %>%
 #remove the canopy size classes:
 filter(SizeClass != "10-20DBH") %>%
 filter(SizeClass != ">20DBH") %>%
 #Set reference values:
 mutate(FireTrt = relevel(FireTrt, ref = "Unburnt"),
 EcoGrp = relevel(EcoGrp, ref = "Eucalypt"))

### Fit negative binomial model

FireEco1 <-
 DataQ2 %>%

#fit each size class as a separate model
 group_by(SizeClass) %>%

 do(model = glmer.nb(Count ~ FireTrt * EcoGrp + (1|Site) + offset(log(PlotSize)),
 data = . ,
 control = glmerControl(optimizer = "bobyqa",
 optCtrl = list(maxfun = 1000000))))

### Test for overdispersion

Overdispersion tests suggest there is no overdispersion (non-significant p values):

ODtest3 <- list()

#for each size class model (1-4):
for (i in 1:4){

#name output
 name <- paste(FireEco1$SizeClass[[i]])

#run overdispersion function
 ODtest3[[name]] <- overdisp_fun(Fire2$model[[i]])
}

ODtest3

## $`<0.5m`
## chisq ratio rdf p
## 15.8044942 1.1288924 14.0000000 0.3254589
##
## $`0.5-1.5m`
## chisq ratio rdf p
## 15.0360441 1.0740031 14.0000000 0.3756952
##
## $`>1.5m`
## chisq ratio rdf p
## 13.9765404 0.9983243 14.0000000 0.4514603
##
## $`5-10DBH`
## chisq ratio rdf p
## 13.8427320 0.9887666 14.0000000 0.4614923

### Fit random slope model

FireEco2 <-
 DataQ2 %>%

#fit each size class as a separate model
 group_by(SizeClass) %>%

 do(model = glmer.nb(Count ~ FireTrt * EcoGrp + (FireTrt|Site) + offset(log(PlotSize)),
 data = . ,
 control = glmerControl(optimizer = "bobyqa",
 optCtrl = list(maxfun = 1000000))))

### Compare models by parametric bootstrap

PBtest2 <- list()

#for each size class model (1-4):
for (i in 1:4) {

#name output

name <- paste(FireEco1$SizeClass[[i]])

#do the model comparison

PBtest2[[name]] <- PBmodcomp(FireEco2$model[[i]], FireEco1$model[[i]], nsim = 1000, seed = 84)
}

PBtest2 output:

## $`<0.5m`
## Parametric bootstrap test; time: 3500.43 sec; samples: 1000 extremes: 190;
## Requested samples: 1000 Used samples: 933 Extremes: 190
## large : Count ~ FireTrt * EcoGrp + (FireTrt | Site) + offset(log(PlotSize))
## small : Count ~ FireTrt * EcoGrp + (1 | Site) + offset(log(PlotSize))
## stat df p.value
## LRT 4.5725 5 0.4702
## PBtest 4.5725 0.2045
##
## $`0.5-1.5m`
## Parametric bootstrap test; time: 2867.86 sec; samples: 1000 extremes: 137;
## Requested samples: 1000 Used samples: 920 Extremes: 137
## large : Count ~ FireTrt * EcoGrp + (FireTrt | Site) + offset(log(PlotSize))
## small : Count ~ FireTrt * EcoGrp + (1 | Site) + offset(log(PlotSize))
## stat df p.value
## LRT 4.824 5 0.4377
## PBtest 4.824 0.1498
##
## $`>1.5m`
## Parametric bootstrap test; time: 4154.01 sec; samples: 1000 extremes: 99;
## Requested samples: 1000 Used samples: 942 Extremes: 99
## large : Count ~ FireTrt * EcoGrp + (FireTrt | Site) + offset(log(PlotSize))
## small : Count ~ FireTrt * EcoGrp + (1 | Site) + offset(log(PlotSize))
## stat df p.value
## LRT 5.2429 5 0.387
## PBtest 5.2429 0.106
##
## $`5-10DBH`
## Parametric bootstrap test; time: 4035.06 sec; samples: 1000 extremes: 302;
## Requested samples: 1000 Used samples: 987 Extremes: 302
## large : Count ~ FireTrt * EcoGrp + (FireTrt | Site) + offset(log(PlotSize))
## small : Count ~ FireTrt * EcoGrp + (1 | Site) + offset(log(PlotSize))
## stat df p.value
## LRT 2.8554 5 0.7223
## PBtest 2.8554 0.3067

Again, there is no evidence that the random slope model is preferred over the random intercept

### Get predictions by parametric bootstrap

We use the model FireEco1 for subsequent predictions.

The size classes are bootstrapped separately due to different plot sizes:

Bootstrap marginal predictions from small and advanced resprout models:

#define new data

ND <- data.frame(expand.grid(FireTrt = levels(DataQ2$FireTrt),
 EcoGrp = levels(DataQ2$EcoGrp),
 PlotSize = 360))

BootFE1 <- NULL

#bootstrap the model
for(i in 1:2) {
 Boot4 <- bootMer(FireEco1$model[[i]], predictFun, nsim = 1000, use.u = FALSE, seed = 84)

 BootFE1 <-
 #bind each model bootstrap outcome together
 bind_rows(BootFE1,
 sumBoot(Boot4) %>%
 mutate(Model = i))
}

Bootstrap marginal predictions from sapling model:

#define new data

ND <- data.frame(expand.grid(FireTrt = levels(DataQ2$FireTrt),
 EcoGrp = levels(DataQ2$EcoGrp),
 PlotSize = 720))

#bootstrap the model
Boot5 <- bootMer(FireEco1$model[[3]], predictFun, nsim = 1000, use.u = FALSE, seed = 84)

 BootFE1 <-
 #bind each model bootstrap outcome together
 bind_rows(BootFE1,
 sumBoot(Boot5) %>%
 mutate(Model = 3))

Bootstrap marginal predictions from pole model:

#define new data

ND <- data.frame(expand.grid(FireTrt = levels(DataQ2$FireTrt),
 EcoGrp = levels(DataQ2$EcoGrp),
 PlotSize = 1800))

#bootstrap the model
Boot6 <- bootMer(FireEco1$model[[4]], predictFun, nsim = 1000, use.u = FALSE, seed = 84)

 BootFE1 <-
 #bind each model bootstrap outcome together
 bind_rows(BootFE1,
 sumBoot(Boot6) %>%
 mutate(Model = 4))

BootFE1 <-
 BootFE1 %>%

#add SizeClass labels by joining the model summary columns
 inner_join(FireEco1 %>%
 rownames_to_column(var = "Model") %>%
 mutate(Model = as.numeric(Model)) %>%
 dplyr::select(-model))
